# Effect of Mn and Fe on the Formation of Fe- and Mn-Rich Intermetallics in Al–5Mg–Mn Alloys Solidified Under Near-Rapid Cooling

**DOI:** 10.3390/ma9020088

**Published:** 2016-01-29

**Authors:** Yulin Liu, Gaoren Huang, Yimeng Sun, Li Zhang, Zhenwei Huang, Jijie Wang, Chunzhong Liu

**Affiliations:** Liaoning Provincial Key Laboratory of Light Alloys and Processing Technology, School of Materials Science and Engineering, Shenyang Aerospace University, Shenyang 110136, China; grhuang2014@sina.com (G.H.); ymengsun@sina.com (Y.S.); zhangli@sau.edu.cn (L.Z.); zwhuang@sau.edu.cn (Z.H.); wangjijie@sau.edu.cn (J.W.); czliu@sau.edu.cn (C.L.)

**Keywords:** aluminum alloy, intermetallics, phase transformation, casting, rapid solidification

## Abstract

Mn was an important alloying element used in Al–Mg–Mn alloys. However, it had to be limited to a low level (<1.0 wt %) to avoid the formation of coarse intermetallics. In order to take full advantage of the benefits of Mn, research was carried out to investigate the possibility of increasing the content of Mn by studying the effect of cooling rate on the formation of Fe- and Mn-rich intermetallics at different content levels of Mn and Fe. The results indicated that in Al–5Mg–Mn alloy with low Fe content (<0.1 wt %), intermetallic Al_6_(Fe,Mn) was small in size and amount. With increasing Mn content, intermetallic Al_6_(Fe,Mn) increased, but in limited amount. In high-Fe-containing Al–5Mg–Mn alloys (0.5 wt % Fe), intermetallic Al_6_(Fe,Mn) became the dominant phase, even in the alloy with low Mn content (0.39 wt %). Cooling rate played a critical role in the refinement of the intermetallics. Under near-rapid cooling, intermetallic Al_6_(Fe,Mn) was extremely refined. Even in the high Mn and/or high-Fe-containing alloys, it still demonstrated fine Chinese script structures. However, once the alloy composition passed beyond the eutectic point, the primary intermetallic Al_6_(Fe,Mn) phase displayed extremely coarse platelet-like morphology. Increasing the content of Fe caused intermetallic Al_6_(Fe,Mn) to become the primary phase at a lower Mn content.

## 1. Introduction

Al–Mg–Mn alloys AA5182 and AA5083 have found great applications in the automotive, marine, packaging, and construction industries due to their special characteristics, such as good weldability, ductility, toughness, formability, and corrosion resistance. In Al–Mg based alloys, Fe was the main impurity and formed the Fe-rich intermetallic, Al_3_Fe, in needle-like form [1]. This type of intermetallic has been considered most detrimental to the mechanical properties of the alloy, due to its brittle features and stress concentration caused by the needle-like morphology [2,3,4]. Fe was also considered a detrimental element in corrosion resistance [5,6]. Mn had usually been added to Al–Mg-based alloys to compensate for the negative effects of the Fe-rich intermetallic Al_3_Fe. The addition of Mn to the alloy transformed the Al_3_Fe phase to the Al_6_(Fe,Mn) phase, and the needle-like morphology of Al_3_Fe to the Chinese script morphology of Al_6_(Fe,Mn) [7,8]. It was thought that intermetallics with Chinese script morphology would have less harmful effects on the mechanical properties of the alloy. In addition, Mn, through solid solution strengthening, could increase the strength of the alloy. Thus, increasing the content of Mn could also increase the strength of the alloy. For example, increasing Mn from 0.35 wt % in the AA5182 alloy to 0.7 wt % in the AA5083 alloy resulted in the increase of tensile strength by 15 MPa at O temper [9]. Therefore, a higher content of Mn favored the strength of the alloy.

On the other hand, with the increase of Mn content, intermetallic Al_6_(Fe,Mn) would also increase in both size and amount. The coarse Al_6_(Fe,Mn) would significantly deteriorate the mechanical properties of the alloy. As a result, increasing the content of Mn affected the alloy in two aspects: increasing its strength due to the increase of Mn atoms in solid solution, and decreasing its strength due to the increase of intermetallic Al_6_(Fe,Mn) in both size and amount. In order to balance the positive and negative effects of Mn, the content of Mn had to be limited to a low level to control the size and amount of intermetallic Al_6_(Fe,Mn). As a result, the benefit of solid solution strengthening of Mn could not be fully utilized. If intermetallic Al_6_(Fe,Mn) could be refined during solidification, its deteriorating effect could be inhibited, then a higher level of Mn could be accepted. Then, more Mn could be added to the alloy to improve its mechanical properties through solid solution strengthening. Finding a solution to refine the intermetallics during solidification was of great commercial interest for the production of the Al–Mg based aluminum alloys.

Cooling rate could greatly influence the intermetallics formed during solidification in terms of size, amount, and morphology. Under near-rapid cooling, the intermetallics could be significantly refined or even inhibited [10]. The cooling rate used in the continuous strip casting (CC) process of aluminum alloy was about 10^1^–10^2^ °C·s^−1^, a near-rapid cooling. Intermetallic Al_6_(Fe,Mn) formed in this process could be significantly refined, and additional Mn could be added without the formation of coarse intermetallic Al_6_(Fe,Mn). Thus, a high-Mn-containing alloy with advanced mechanical properties could be developed and produced by using the CC process. The CC process has the advantage of high productivity and low production cost and has recently received wide attention from manufacturers and end users [11]. It was necessary and important to conduct research to study high-Mn-containing Al–Mg–Mn alloys solidified under near-rapid cooling.

Extensive research had been carried out on the Al–Mg–Mn alloys, and a large number of investigations focused on microstructures and properties [12,13,14], superplasticity [15,16,17], ultrafine grain [18,19,20,21,22], and adding element [23,24,25,26], *etc.* Al–Mg–Mn aluminum alloy sheet materials produced using the CC process have also been widely investigated [27,28,29,30,31]. However, little investigation has been made on the formation and control of the intermetallics in high-Mn-containing Al–Mg–Mn alloy solidified under near-rapid cooling. 

This work investigated the intermetallics formed during solidification and the influence of Mn, Fe, and cooling rate on the formation of intermetallic Al_6_(Fe,Mn). Their effect on the microstructures and mechanical properties of Al–5Mg–Mn alloys solidified under near-rapid cooling was reported elsewhere. The research simulated the CC process. The ultimate aim was to develop and produce a high-Mn-containing Al–Mg alloy with higher mechanical properties using the high productivity and low-cost CC process.

## 2. Experimental Procedures

**Figure 1:**
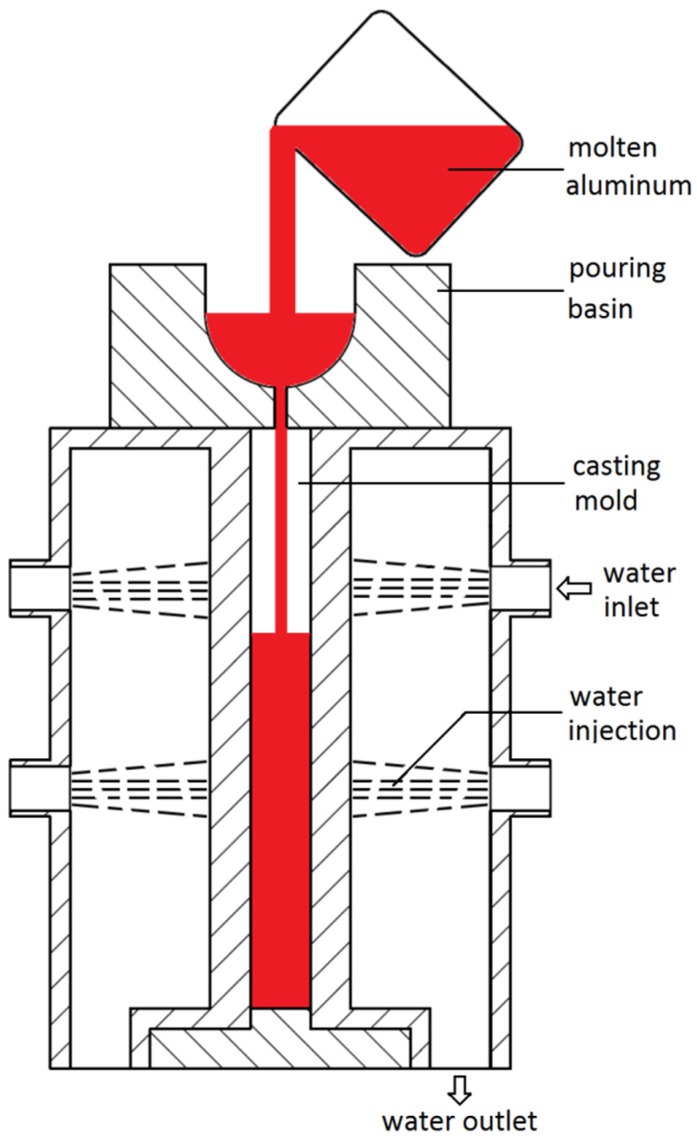
Schematic sketch of the double-side water cooling casting apparatus.

A double-side water-cooled casting apparatus was designed to simulate the cooling of the CC process, as shown in Figure 1. The casting mold was 150 mm × 150 mm × 15 mm in size and was continuously cooled from two sides by injecting cold water. The cooling rate during casting was about 20 °C·s^−1^, measured by recording the temperature evolution with time.

Two groups of Al–5Mg–Mn alloys were prepared. In each group, five alloys were designed with different Mn content, varying from 0.4 wt % to 2.0 wt %. In the Group I alloys, the contents of iron and silicon were low (<0.1 wt % Fe and <0.05 wt % Si). Higher contents of Fe and Si (0.5 wt % Fe and 0.25 wt % Si) were used in the Group II alloys to study the combined effect of Mn, Fe, and Si. 

High grade commercial pure aluminum ingots with less than 0.10 wt % iron and less than 0.04 wt % silicon, magnesium ingots (99.9 wt %), and Al–10Fe and Al–30Si master alloys were used to prepare the studied alloys. For each designed alloy, 2 kg of raw materials were melted in a graphite-clay crucible in an electric resistance furnace. After being degassed by injecting Ar into the pool and refined by adding Al–5Ti–B grain refiner, the molten aluminum, at around 725 °C, was then poured into the apparatus shown in Figure 1. The chemical compositions of the cast slabs were determined by emission spectroscopy and listed in Table 1.

**Table 1:** Chemical composition of alloy samples, wt %. Bal. means balance.

Group	Alloy Sample	Nominal Content of Mn	Measured Composition						
			Mg	Mn	Fe	Si	Cr	Ti	Al
I	I-1	0.4	4.75	0.39	0.085	0.034	0.006	0.018	Bal.
	I-2	0.8	5.19	0.80	0.092	0.035	0.006	0.016	Bal.
	I-3	1.2	5.08	1.29	0.085	0.033	0.006	0.016	Bal.
	I-4	1.6	4.83	1.63	0.089	0.036	0.006	0.017	Bal.
	I-5	2.0	5.13	2.10	0.098	0.034	0.006	0.017	Bal.
II	II-1	0.4	4.81	0.39	0.455	0.233	0.006	0.017	Bal.
	II-2	0.8	4.87	0.73	0.485	0.241	0.006	0.023	Bal.
	II-3	1.2	4.82	1.23	0.473	0.219	0.006	0.018	Bal.
	II-4	1.6	5.12	1.51	0.502	0.238	0.006	0.022	Bal.
	II-5	2.0	4.83	2.00	0.556	0.230	0.006	0.024	Bal.

In order to compare the as-cast microstructure of the alloys solidified under slow cooling, samples were cut from each cast slab and were re-melted and then solidified in alumina crucibles in an electric resistance furnace. The temperature evolutions with time were recorded. The average cooling rate before the start of solidification was approximately 0.065 K s^−1^ (°C·s^−1^), which was calculated by using the formula d*T*/d*t* and computed from the approximate straight line portion of the cooling curve. 

A series of interrupted water quenchings were conducted to reveal the solidification sequences of the alloy. During solidification, the temperature of the molten samples was monitored in-line by inserting the thermocouple into the molten samples. Once the predetermined temperature was reached, the solidification process was interrupted and the solidifying sample was quenched into cold water. The solidification process of the alloy could be revealed by differentiating between the solidified structure and the water-quenched structure of the samples. This method had been proven to work by the present author’s previous work [32] and recently the work of Liu *et al.* [33].

An OLYMPUS GX71 optical microscope (OM) from Olympus Corporation (Shinjuku, Japan), a Zeiss scanning electron microscopy (SEM) with energy dispersive X-ray (EDX) analyzer from Zeiss Group (Oberkochen, Germany) and a X’pert Pro X-ray diffractometer (XRD) from PANalytical (Almelo, The Netherlands) were employed to analyze the intermetallics formed during solidification. The metallographic samples were prepared using standard metallographic procedures and electropolished at a voltage of 27 V DC for 15 s using 60 mL HClO_4_ + 140 mL H_2_O + 800 mL C_2_H_5_OH. 

## 3. Results

### 3.1. Intermetallics Formed under Near-Rapid Cooling

**Figure 2:**
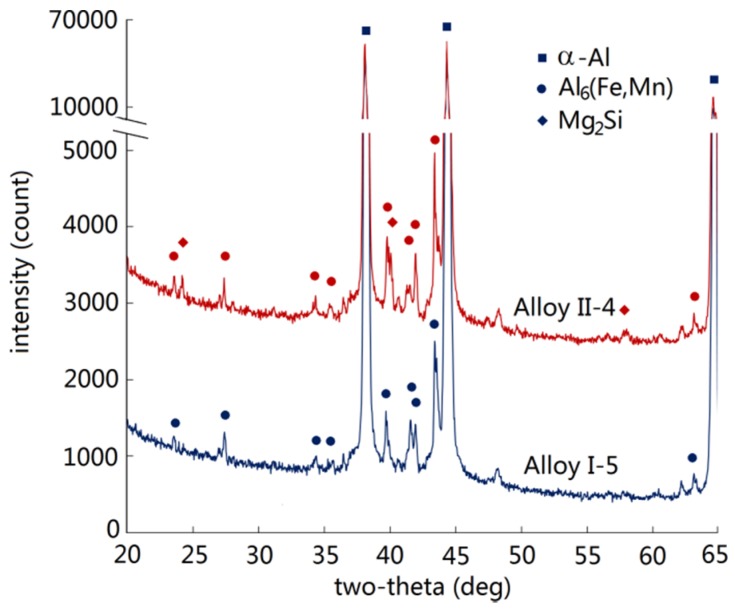
The XRD spectra of Alloy I-5 and Alloy II-4.

The as-cast microstructures of the alloys cast under near-rapid cooling (approximately 20 °C·s^−1^) were identified by XRD analysis. They were affected by the contents of Mn, Fe, and Si. The XRD spectra of the Group I alloys were very simple. In the low-Mn alloys (Alloy I-1 and Alloy I-2), only α-Al was detected, which was the matrix of the alloys. With the increase of Mn content in the alloys, the spectrum of intermetallic Al_6_(Fe,Mn) gradually became visible. It was relatively strong in Alloy I-5. In the Group II alloys, the spectra of intermetallic Al_6_(Fe,Mn) was already quite strong in Alloy II-1 with low Mn content and increased with increasing Mn content. Apart from Al_6_(Fe,Mn), intermetallic Mg_2_Si was also detected. XRD spectra showed the same mode in all the Group II alloys. Figure 2 shows the XRD spectra of two typical alloys. Two intermetallics Al_6_(Fe,Mn) and Mg_2_Si were read from the XRD analyses.

**Figure 3:**
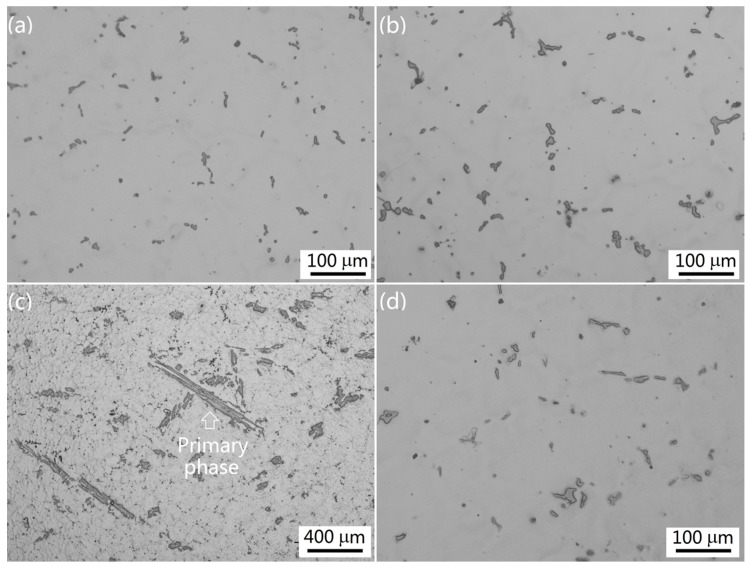
The as-cast microstructures of the Group I alloys solidified under near-rapid cooling: (**a**) Alloy I-1; (**b**) Alloy I-4; (**c**,**d**) Alloy I-5.

Figure 3 shows the as-cast microstructures of some typical Group I alloys solidified under near-rapid cooling. They consisted of α-Al matrix and intermetallics. The intermetallic structures were quite simple: faceted, with a small blocky morphology. It was found by careful observation that they consisted of a major phase in light gray and a minor phase in dark gray. However, they were hard to distinguish in the metallographic photos. In Alloy I-1, the intermetallics were rare and small (Figure 3a). With the increase of Mn content in the alloys, the size and amount of the major intermetallic increased correspondingly. However, the increment was not remarkable. Even in Alloy I-4 with 1.6 wt % Mn, the intermetallic was not significantly increased (Figure 3b). Significant change of the major intermetallic occurred in Alloy I-5, with 2.0 wt % Mn (Figure 3c,d). In this sample, some extremely large platelet-like intermetallic was observed (Figure 3c). The intermetallic displayed a mixed structure of coarse platelet-like, small blocky, and Chinese script. The eutectic intermetallics were similar to the ones observed in Alloy I-4 (Figure 3d). In all the Group I alloys, the minor intermetallic was almost the same: very small in both size and amount. 

**Figure 4:**
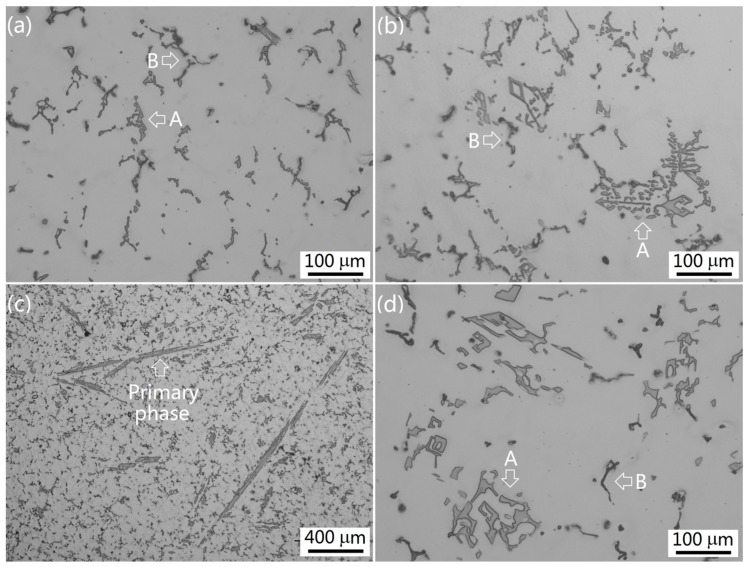
The as-cast microstructures of the Group II alloys solidified under near-rapid cooling (**a**) Alloy II-1; (**b**) Alloy II-3; (**c**,**d**) Alloy II-4.

In the Group II alloys with high contents of Fe and Si, both the major intermetallic and the minor intermetallic significantly increased in terms of size and amount. They could be easily distinguished on the metallographic photos and were marked as A and B, respectively. Alloy II-1 displayed a large amount of the major intermetallic in Chinese script morphologies. The minor phase also showed Chinese script morphology, but was in a smaller amount than the major phase (Figure 4a). Compared to Alloy I-1, the as-cast microstructures significantly changed in Alloy II-1, as Fe and Si also participated in the formation of the intermetallics. As in the Group I alloys, increasing the Mn content in the Group II alloys also resulted in a corresponding increase in the size and amount of the major intermetallic. However, no change occurred to the minor phase in terms of size and amount. In Alloy II-3, some intermetallic became a flower-like structure. In addition, massive blocky or diamond shapes were also observed, as shown in Figure 4b. An extremely coarse platelet-like intermetallic appeared in Alloy II-4 which had a lower content of Mn than Alloy I-5. Most likely, in the alloys with a high Fe content, an extremely coarse platelet-like intermetallic formed at a relatively low content of Mn. In Alloy II-5 with 2.0 wt % Mn, the size and amount of the coarse platelet-like and diamond-like phases further increased. The morphology, distribution, size, and amount of the minor phase were almost the same in all of the Group II alloys. 

**Figure 5:**
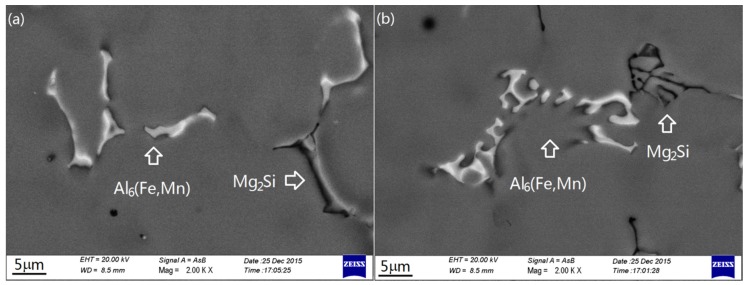
High magnification images of the intermetallics (**a**) Alloy I-4; (**b**) Alloy II-1.

The intermetallics were further studied by using SEM/EDX. Figure 5 shows the SEM backscattered electronic images of the typical morphologies of the major intermetallic and minor intermetallic at high magnification. Table 2 lists the results of the EDX analysis. It was found that, in both Group I and Group II alloys, the major intermetallic was Fe- and Mn-rich intermetallic; the minor intermetallic mainly contained Mg and Si. The composition of the former was close to the formula Al_6_(Fe,Mn), but the composition of the latter did not meet the formula of any known phase. With the increase of Mn content, the composition formula of the major intermetallic was even closer to Al_6_(Fe,Mn); the concentration of Fe in the phases decreased slightly. The extremely coarse platelet-like intermetallic in Alloy I-5 had the same composition as the major intermetallic in the previous alloys. No remarkable change in composition was detected in the Mg- and Si-containing phase among the alloys. 

**Table 2:** Composition of intermetallic Al_6_(Fe,Mn) measured by EDX (at%).

Element	Sample I-4		Sample I-5	
	Composition Range	Composition Average	Composition Range	Composition Average
Al	76.32–85.19	80.46	83.41–86.00	84.82
Mn	9.36–17.91	14.96	13.37–15.74	14.38
Fe	1.88–3.35	2.9	0.63–0.91	0.80
Mg	0–3.57	1.71	0	0

The content of Fe had great influence on the major intermetallic. Although the composition of the major intermetallic in the Group II alloys were also close to the formula Al_6_(Fe,Mn), the concentration of Fe significantly increased and became the major element in the phase (Table 3). However, increasing Si in the Group II alloys did not change the composition of the Si- and Mg-containing phase. They still deviated from the stoichiometric ratio of Mg_2_Si.

**Table 3:** Composition of intermetallic Al_6_(Fe,Mn) measured by EDX (at%).

Element	Alloy II-1		Alloy II-4	
	Composition Range	Composition Average	Composition Range	Composition Average
Al	85.62–86.03	85.83	81.08–82.25	81.83
Mn	2.17–2.34	2.27	12.08–13.53	12.82
Fe	10.58–11.35	10.95	4.98–5.67	5.35
Mg	0.74–1.09	0.81	0	0

According to the analyses of XRD, SEM/EDX, and metallographic observation, it was certain that in all the alloys the major intermetallic was the Al_6_(Fe,Mn) phase. The extremely coarse platelet-like intermetallic in Alloys I-5, II-4 and II-5 was also Al_6_(Fe,Mn). Most likely, they precipitated as the primary phase during solidification. Although the EDX-measured composition of the minor intermetallic did not meet the stoichiometric formula of Mg_2_Si, considering the fact that the Mg_2_Si spectrum was detected by XRD, most likely, this minor intermetallic was the Mg_2_Si phase. The aluminum detected in the phase was from the matrix.

**Figure 6:**
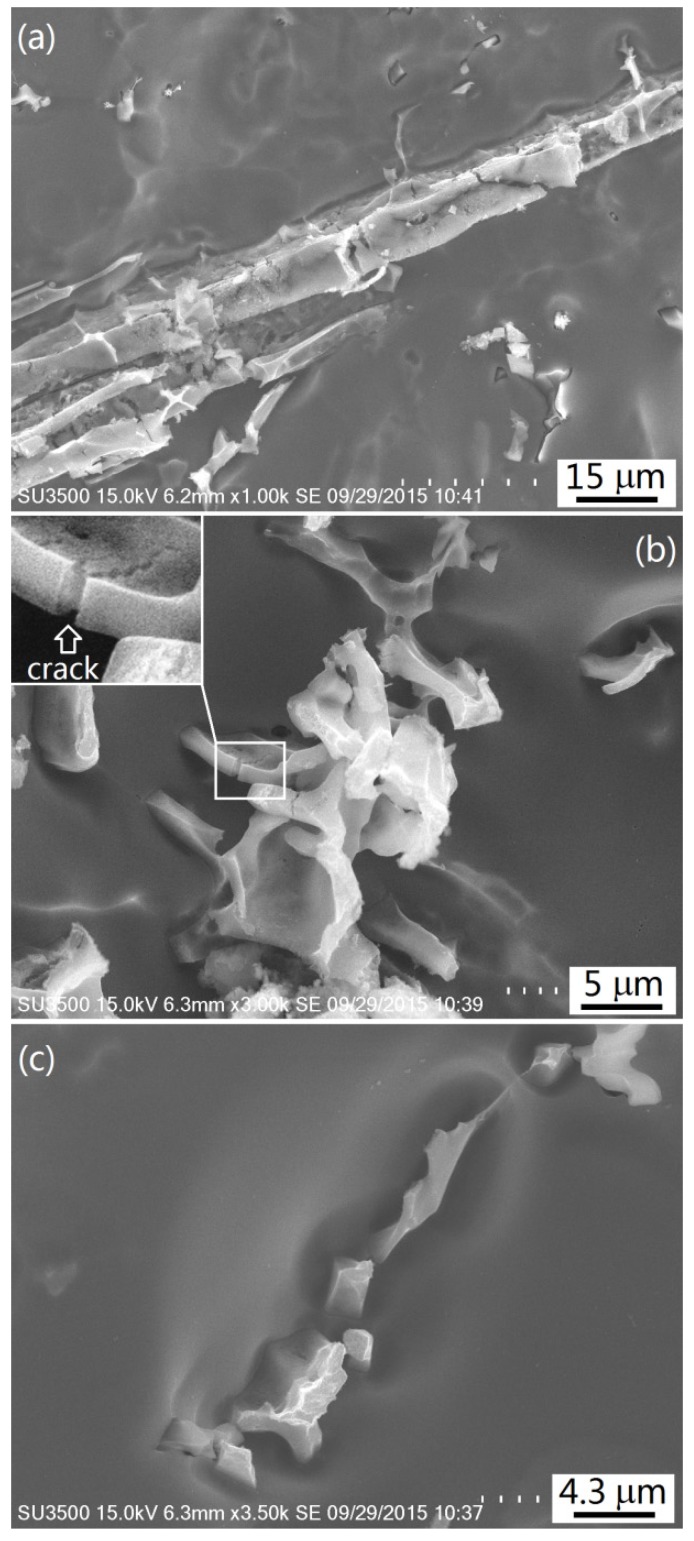
The deep etched images of the intermetallics: (**a**) Primary Al_6_(Fe,Mn); (**b**) eutectic Al_6_(Fe,Mn); (**c**) Mg_2_Si.

Figure 6 shows the deep-etched images of the Alloy II-5 intermetallics. After deep-etching with NaOH water solution for 1 min, some square-pillar like structure appeared. It could be seen that the platelet-like and square shapes observed under OM (Figure 4c) were the longitudinal and transversal cross sections of the square pillar, respectively. The complicated structure with a coral-like morphology should correspond to the Chinese script-like Al_6_(Fe,Mn). The Mg_2_Si phase was relatively simple with a small blocky structure (Figure 6c). In addition, it was worth noting that some microcracks appeared on the square pillar and coral-like Al_6_(Fe,Mn), especially present in the square pillar.

### 3.2. Intermetallics Formed under Slow Cooling

**Figure 7:**
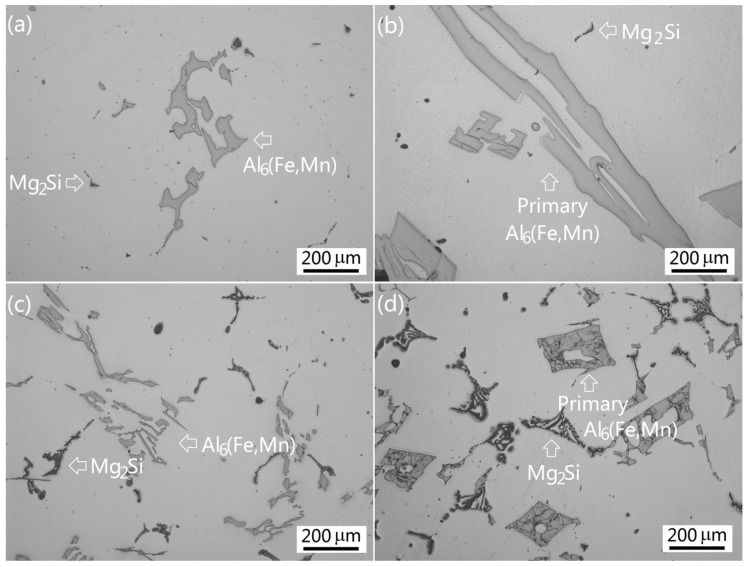
The as-cast microstructures of the alloys solidified under slow cooling (**a**) Alloy I-4; (**b**) Alloy I-5; (**c**) Alloy II-1; (**d**) Alloy II-4.

In order to compare the microstructure of the near-rapid cooled alloys with the direct-chill (DC) casting process cooled alloys, the alloys were remelted and solidified at a cooling rate of approximately 0.065 °C·s^−1^, which simulated the solidification of DC cast ingot. It was found that the as-cast microstructures of the alloys were essentially the same as described above. They still consisted of α-Al matrix and intermetallics Al_6_(Fe,Mn) and Mg_2_Si. However, their size, amount, morphologies, and distribution were completely different, especially in the high-Mn alloys. In the Group I alloys, intermetallic Al_6_(Fe,Mn) of Alloy I-1 was very small in both size and amount and appeared to be quite faceted with a blocky morphology. With the increase of Mn content in the alloy, intermetallic Al_6_(Fe,Mn) gradually increased in size and amount. In Alloy I-3 with 1.2 wt % Mn, intermetallic Al_6_(Fe,Mn) branched. Increasing the Mn content to 1.6 wt % (Alloy I-4), the morphologies of Al_6_(Fe,Mn) became complicated with flower-like or Chinese script shapes (Figure 7a). In Alloy I-5 with 2.0 wt % Mn, the Al_6_(Mn,Fe) phase increased significantly in both amount and size. A large amount of massive platelet-like Al_6_(Fe,Mn) was observed (Figure 7b). Intermetallic Mg_2_Si showed irregular morphologies and were very small in both size and amount in all the Group I alloys.

The solidification structures of the Group II alloys still consisted of α-Al matrix and intermetallics Al_6_(Fe,Mn) and Mg_2_Si. In Alloy II-1, Al_6_(Fe,Mn) turned into complicated flower-like structures in large sizes; Mg_2_Si turned into network and Chinese script structures (Figure 7c). With the increase of Mn content, the amount and size of Al_6_(Fe,Mn) increased accordingly. Figure 7d shows the extremely large square-shaped primary Al_6_(Fe,Mn) in Alloy II-4. The as-cast microstructure was extremely coarse. In Alloy II-5, intermetallic Al_6_(Fe,Mn) further increased in both size and amount.

### 3.3. The Solidification Process

**Figure 8:**
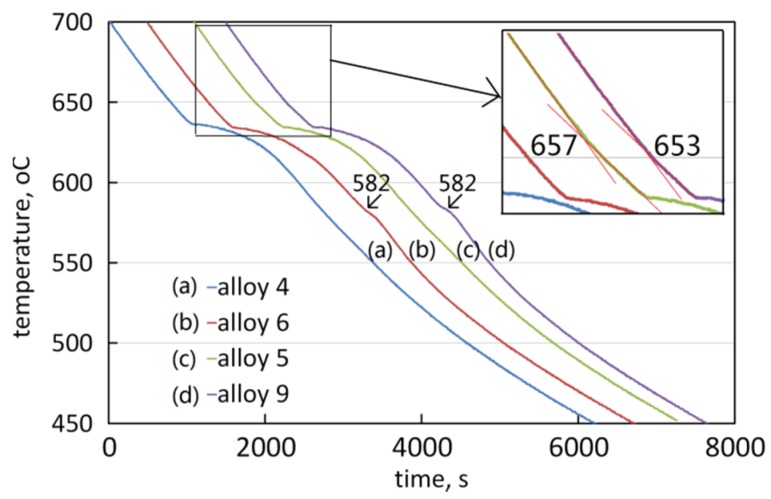
The cooling curves of the alloys.

Figure 8 shows the cooling curves of the four typical alloys during solidification at a cooling rate of approximately 0.065 °C·s^−1^. It can be seen that all the alloys had an inflexion at around 634 °C. These inflexion points correspond to the start of solidification of the α-Al matrix. In addition, on the curves of Alloy I-5 and Alloy II-4, an inflexion was detected at 657 °C and 653 °C, respectively. On the curves of Alloy II-1 and Alloy II-4, an inflexion was detected at 582 °C. It was assumed that the inflexions at 657 °C and 653 °C corresponded to the precipitation of the primary intermetallic Al_6_(Fe,Mn); the inflexion at 582 °C corresponded to the precipitation of the intermetallic Mg_2_Si.

**Figure 9:**
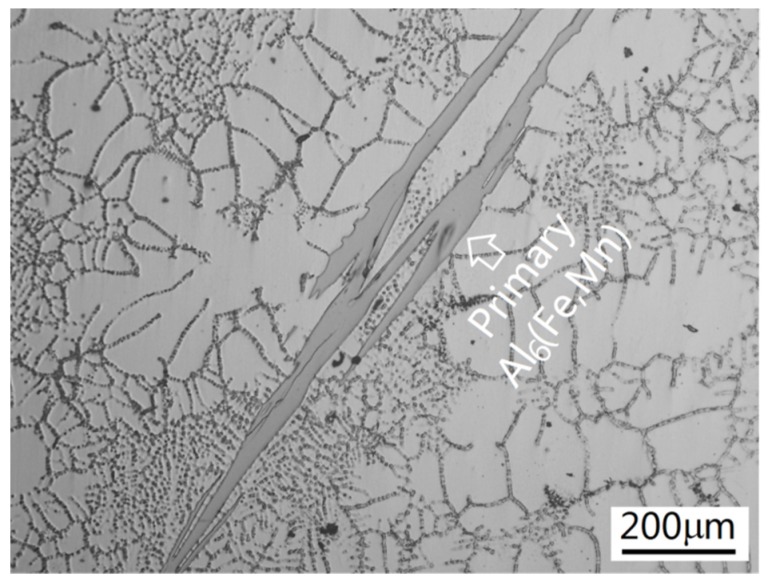
The microstructures of Alloy I-5 after interrupted quenching at 650 °C.

In order to confirm the precipitation of the primary phase, a series of interrupted water quenchings was performed during the cooling of Alloy I-4 and Alloy I-5. When Alloy I-4 cooled to 630 °C, which was lower than the assumed precipitation temperature of α-Al, the cooling sample was quenched into cold water. In the quenched sample, no intermetallic Al_6_(Fe,Mn) was discovered. This confirmed that the primary phase was α-Al. Alloy I-5 was quenched into cold water when it cooled to 650 °C, which was much higher than the assumed precipitation temperature of α-Al. Intermetallic Al_6_(Fe,Mn) had already precipitated in the quenched sample, see Figure 9. These experiments confirmed that Alloy I-4 was still hypoeutectic, α-Al was the primary phase. However, Alloy I-5 was hypereutectic, intermetallic Al_6_(Fe,Mn) became the primary phase. These results agreed with the deduction mentioned above.

## 4. Discussion

The as-cast intermetallic structures were significantly affected by the contents of Mn and Fe and cooling rate in terms of size, amount, morphology, and distribution.

### 4.1. The Influence of Mn and Fe

According to the Al–Mn binary diagram [34], the redistribution coefficient of Mn in Al was about 0.95. Therefore, the segregation tendency of Mn was quite small. According to the Scheil equation:

*C_L_ = C*_0_·*f_L_^(k−^*^1*)*^ (1)

where *k* is the redistribution coefficient of Mn*, C*_0_ is the initial concentration of Mn in the alloy, *C_L_* is the concentration of Mn in the liquid in interface front , and *f_L_* is the fractional volume of remaining liquid.

During solidification, the buildup of Mn concentration in the interface front was very limited. Most of the Mn atoms were in solid solution in the matrix. In the alloy with low Mn content, the final solidification zones that reached the eutectic composition were limited in both size and number. Therefore, the intermetallics formed during solidification were small in both size and amount. With the increase of Mn content in the alloys, the concentration of Mn at the interface front increased, which resulted in the increase in the number of final solidification zones that reached eutectic composition. However, according to Equation (1), the increment was limited. Therefore, increasing the Mn content in the alloys increased the amount of Mn atoms in solid solution in the matrix, but did not significantly increase the fraction of eutectic intermetallics. If the eutectic intermetallics could be refined to avoid the deteriorating effect of the coarse intermetallic, increasing Mn content in the alloys could result in the increase of strength by solution strengthening. This favors improving the mechanical properties of the alloy by increasing the Mn content.

On the other hand, once the Mn content passed beyond the eutectic point, intermetallic Al_6_(Fe,Mn) became the primary phase, then the eutectic reaction L → α-Al + Al_6_(Fe,Mn) followed. Therefore, almost all of the Mn atoms would form intermetallic Al_6_(Fe,Mn), which was large in both size and amount. The amount of Mn atoms in solid solution was limited. In this case, increasing Mn content in the alloys would result in the deterioration of the mechanical properties.

From the Al–Fe phase diagram [35], the redistribution coefficient of Fe in Al was about 0.022. Therefore, the segregation tendency of Fe was very high. Almost all the Fe segregated into the interface front and formed Fe-rich intermetallic during solidification. Increasing the content of Fe in the alloy would significantly increase the volume fraction of the Fe-rich intermetallic. In Al–Mg alloys, for example the AA5005 alloy, Fe combined with Al to form Al_3_Fe [1]. Adding Mn gave rise to the formation of Fe- and Mn-rich intermetallic. Early work [36] showed that the three phases Al_6_Mn, Al_6_(Mn,Fe), and Al_6_Fe were isomorphous. In the alloy system that contained both Mn and Fe, Fe combined with Mn to form Fe- and Mn-rich intermetallic Al_6_(Fe,Mn). The amount of Al_6_(Fe,Mn) formed during solidification depended on the contents of both Mn and Fe. In the alloys with low Fe content, increasing the content of Mn to 1.6 wt % did not cause the formation of primary Al_6_(Fe,Mn). The amount of intermetallic Al_6_(Fe,Mn) formed was quite small. However, in the high Fe alloy, Fe caused the formation of a large amount of intermetallic Al_6_(Fe,Mn). Increasing Mn would give rise to the further increase of the intermetallic. Therefore, there existed a large amount of intermetallic Al_6_(Fe,Mn) in all the Group II alloys. Since both Mn and Fe contributed to the formation of intermetallic Al_6_(Fe,Mn), the eutectic composition of Mn for the reaction of L → α-Al + Al_6_(Fe,Mn) would be lower than for the reaction of L → α-Al + Al_6_Mn. Therefore, primary intermetallic Al_6_(Fe,Mn) was observed in Alloy II-4. As a result, the limit of Mn in the alloy composition specification had to be reduced in order to avoid the formation of coarse primary intermetallic Al_6_(Fe,Mn).

In this study, Si combined with Mg to form the Mg_2_Si phase. No AlFeSi or AlFeMnSi phase was observed. On the other hand, Si also had a great influence on the Fe- and Mn-rich intermetallics. A research on Al–5Mg–0.8Mn alloy with 0.5 wt % Fe and 0.5 wt % Si indicated that the Si-containing intermetallic would change to the quaternary phase Al_15_(Fe,Mn)_3_Si with fine fish bone or Chinese script structure when the alloy was solidified under near-rapid cooling [37].

### 4.2. The Influence of Cooling Rate

Comparing the intermetallic structures of the alloys formed under near-rapid cooling and slow cooling, it could be found that cooling rate played a key role in the control of the intermetallic structures. Increasing the cooling rate to 20 °C·s^−1^, the intermetallics were extremely refined. Even in the alloy with high content of Fe and Mn, the intermetallics were still small in size. 

During solidification, with the growth of primary α-Al, the solutes Mn and Fe segregated into and enriched at the interface front. Once the composition of the remaining liquid in the final solidification zones reached the eutectic composition, the eutectic reaction L → α-Al + Al_6_(Fe,Mn) occurred. Increasing cooling rate during solidification would result in the decrease of the segregation tendency of the solute atoms and grains. Therefore, the higher the cooling rate, the less the solute atoms segregated into interface front. On the other hand, the decrease of the grain sizes affected the refinement of the intermetallics from two aspects. In general, the final solidification zones were the regions with triangle grain boundaries and inter-dendrites. The refinement of the grains resulted in the increase of regions with triangle grain boundaries and inter-dendrites (final solidification zones) and the decrease of the concentration of solute atoms at the interface front. As a result, the regions in which the concentration of solutes reached the eutectic point increased in amount and decreased in size. The intermetallics formed in the final solidification zones were extremely refined.

Mn had been added to Al–Mg-based alloys to compensate for the negative effect of Fe on the mechanical properties and achieve solid solution strengthening. Increasing the content of Mn would increase the mechanical properties of the Al–Mg-based alloy by increasing solid solution strengthening. For example, tensile strength of the Al–5Mg–Mn alloy at O temper increased 15 MPa when increasing the Mn content from 0.35 wt % (AA5082 alloy) to 0.7 wt % (AA5083 alloy). However, further increase of Mn would cause the formation of coarse intermetallic Al_6_(Fe,Mn) during solidification, as observed in Alloy II-3, and would lead to significant degradation of the alloy’s mechanical properties [2,3]. The deteriorating effect of the intermetallics on the mechanical properties of the alloys was related to the size, amount and morphology of the intermetallics. The coarse intermetallics would break into coarse particles with sharp edges and corners during hot and cold rolling and leave cavities among the fragments. These coarse particles and cavities were very harmful to the mechanical properties. The higher the content of Mn in the alloy, the coarser the intermetallics formed during solidification, and the coarser the particles remained in the hot band and cold rolled sheet. The solidification process under slow cooling in this study was similar to the situation of traditional direct chill casting. Therefore, in the traditional production practice, the concentration of Mn must be limited to a low level in order to avoid the formation of coarse manganese-rich constituents. The solid solution strengthening effect of Mn could not be fully used. 

On the other hand, the intermetallics could be significantly refined by increasing cooling rate during solidification. As mentioned above, intermetallic Al_6_(Fe,Mn) was extremely refined by the near-rapid cooling during solidification (Figure 3 and Figure 4). It was assumed that the particles created from those intermetallics during hot rolling and/or cold rolling would be fine and possess less deteriorating effect on the mechanical properties. As a result, under the condition of near-rapid cooling, the content of Mn in the alloys could be increased to a higher level and fully utilize the advantage of solid solute strengthening of Mn. 

The near-rapid cooling used in this study was comparable to the cooling in the continuous strip casting process. For the production of Al–Mg-based alloy sheets via the CC process, most likely, the limits of Mn could be increased to a higher level to achieve improved mechanical properties.

## 5. Conclusions

1) In Al–5Mg–Mn alloy with low Fe content, intermetallic Al_6_(Fe,Mn) was small in size and amount. With the increase of Mn, intermetallic Al_6_(Fe,Mn) increased, but the increment was limited. Once the content of Mn passed beyond eutectic, intermetallic Al_6_(Fe,Mn) became the primary phase with extremely coarse platelet-like morphology and increased significantly in amount;
2) In high-Fe-containing Al–5Mg–Mn alloys, Fe promoted the formation of intermetallic Al_6_(Fe,Mn). Even in the alloy with low Mn content, it was in a large amount. With the increase of Mn content, intermetallic Al_6_(Fe,Mn) increased in both size and amount. Increasing the content of Fe caused intermetallic Al6(Fe,Mn) to become the primary phase at a lower Mn content;
3) Cooling rate played a critical role in the refinement of intermetallics. The Al_6_(Fe,Mn) phase could be refined to a significant extent by casting under near-rapid cooling (around 20 °C·s^−1^). In the alloys with high Mn and/or high Fe contents solidified under near-rapid cooling, intermetallic Al_6_(Fe,Mn) demonstrated fine Chinese script structures, which could be achieved using the continuous strip casting process.
